# Blood pressure, brain lesions and cognitive decline in patients with atrial fibrillation

**DOI:** 10.3389/fcvm.2024.1449506

**Published:** 2024-09-03

**Authors:** Désirée Carmine, Stefanie Aeschbacher, Michael Coslovsky, Elisa Hennings, Rebecca E. Paladini, Raffaele Peter, Melanie Burger, Tobias Reichlin, Nicolas Rodondi, Andreas S. Müller, Peter Ammann, Giulio Conte, Angelo Auricchio, Giorgio Moschovitis, Julia B. Bardoczi, Annina Stauber, Maria Luisa De Perna, Christine S. Zuern, Tim Sinnecker, Patrick Badertscher, Christian Sticherling, Leo H. Bonati, David Conen, Philipp Krisai, Stefan Osswald, Michael Kühne

**Affiliations:** ^1^ Cardiovascular Research Institute Basel, University Hospital Basel, Basel, Switzerland; ^2^ Department of Cardiology/Electrophysiology, Department of Medicine, University Hospital Basel, University of Basel, Basel, Switzerland; ^3^ Clinical Trial Unit Basel, Department of Clinical Research, University Hospital Basel, Basel, Switzerland; ^4^ Department of Cardiology, Inselspital, Bern University Hospital, University of Bern, Bern, Switzerland; ^5^ Institute of Primary Health Care (BIHAM), University of Bern, Bern, Switzerland; ^6^ Department of General Internal Medicine, Inselspital, Bern University Hospital, University of Bern, Bern, Switzerland; ^7^ Department of Cardiology, Triemli Hospital Zürich, Zürich, Switzerland; ^8^ Department of Cardiology, Cantonal Hospital St. Gallen, St. Gallen, Switzerland; ^9^ Division of Cardiology, Ente Ospedaliero Cantonale (EOC), Cardiocentro Ticino Institute, Regional Hospital of Lugano, Lugano, Switzerland; ^10^ Department of Neurology and Stroke Center, University Hospital Basel, University of Basel, Basel, Switzerland; ^11^ Medical Image Analysis Center (MIAC) and Department of Biomedical Engineering, University of Basel, Basel, Switzerland; ^12^ Research Department, Reha Rheinfelden, Rheinfelden, Switzerland; ^13^ Population Health Research Institute, McMaster University, Hamilton, ON, Canada

**Keywords:** atrial fibrillation, blood pressure, hypertension, brain lesions, cognitive decline

## Abstract

**Background:**

The influence of atrial fibrillation (AF) and blood pressure (BP) on brain lesions and cognitive function is unclear. We aimed to investigate the association of BP with different types of brain lesions and cognitive decline in patients with AF.

**Methods:**

Overall, 1,213 AF patients underwent standardized brain magnetic resonance imaging at baseline and after 2 years, as well as yearly neurocognitive testing. BP was measured at baseline and categorized according to guidelines. New lesions were defined as new or enlarged brain lesions after 2 years. We defined cognitive decline using three different neurocognitive tests. Logistic and Cox regression analyses were performed to examine the associations of BP with new brain lesions and cognitive decline.

**Results:**

The mean age was 71 ± 8.4 years, 74% were male and mean BP was 135 ± 18/79 ± 12 mmHg. New ischemic lesions and white matter lesions were found in 5.4% and 18.4%, respectively. After multivariable adjustment, BP was not associated with the presence of new brain lesions after 2 years. There was no association between BP and cognitive decline over a median follow-up of 6 years when using the Montreal Cognitive Assessment or Digit Symbol Substitution Test. However, BP categories were inversely associated with cognitive decline using the Semantic Fluency Test, with the strongest association in patients with hypertension grade 1 [Hazard Ratio (95% Confidence Interval) 0.57(0.42 to 0.77)], compared to patients with optimal BP (p for linear trend: 0.025).

**Conclusions:**

In a large cohort of AF patients, there was no association between BP and incidence of brain lesions after 2 years. Also, there was no consistent association between BP and cognitive decline over a follow-up of 6 years.

**Clinical Trial Registration:**

https://clinicaltrials.gov/study/NCT02105844, Identifier (NCT02105844).

## 1 Introduction

The global number of individuals affected by atrial fibrillation (AF) and arterial hypertension is continuously increasing and is expected to further rise due to increasing life expectancy (1, 2). AF and hypertension often coexist. First, they are both associated with age (3, 4). Second, elevated blood pressure (BP) levels contribute to a higher risk of developing left ventricular hypertrophy and left atrial dilatation, which are predisposing conditions for AF (5, 6).

In comparison to the general population, patients with AF face an elevated risk of clinical stroke, covert brain lesions and dementia (7–11), which is responsible for years lived with disability and causes important costs to society (12, 13). Within the Swiss Atrial Fibrillation study (Swiss-AF), we identified a high prevalence of ischemic brain infarcts, white matter lesions (WML), and microbleeds (Mb) (11). Moreover, we demonstrated a relevant incidence of new brain lesions over a period of 2 years, despite a high rate of oral anticoagulation (14).

Hypertension, ischemic brain lesions, and WML have been shown to be associated with impaired cognitive function in the general population (15). In patients with AF, a cross-sectional analysis within the Swiss-AF study described an association between hypertension and the presence of different types of brain lesions, mainly WML (16).

A previous study, which is based on health insurance data, investigated time-updated BP levels and risk of dementia in midlife patients with AF and suggests that BP of 120–129/80–84 mm Hg might reduce the risk of dementia (17). Furthermore, they assume that lower BP might increase the risk of Alzheimer dementia, and higher BP might promote vascular dementia (17).

Considering the high burden of both AF and hypertension, and the increasing global prevalence of dementia (12), a better understanding of their association is of high relevance. We therefore aimed to investigate the influence of BP levels and hypertension on incidence, type, and volume of new brain lesions in AF patients after a 2-year follow-up. Furthermore, we aimed to evaluate the association between BP levels and cognitive decline over a follow-up period of up to 8 years.

## 2 Methods

### 2.1 Study design and patient population

This analysis was conducted using data from the ongoing, prospective, multicenter Swiss-AF study. Patients aged ≥65 years with documented AF were enrolled in 14 different centers across Switzerland between 2014 and 2017. Additionally, a smaller sample (10%) of patients aged between 45 and 65 years was also included to study socioeconomic aspects. The detailed study design was published previously (ClinicalTrials.gov: NCT02105844) (18). Exclusion criteria were the lack of a written informed consent, short transient forms of AF due to reversible causes (e.g., surgery or infection) and the presence of any acute disease in the previous month. At baseline, all patients had a personal visit at their respective study center. Yearly follow-up visits were done either personally or by phone. The Swiss-AF study follows the Declaration of Helsinki, and the protocol was accepted by the responsible ethics committees.

**Figure 1:**
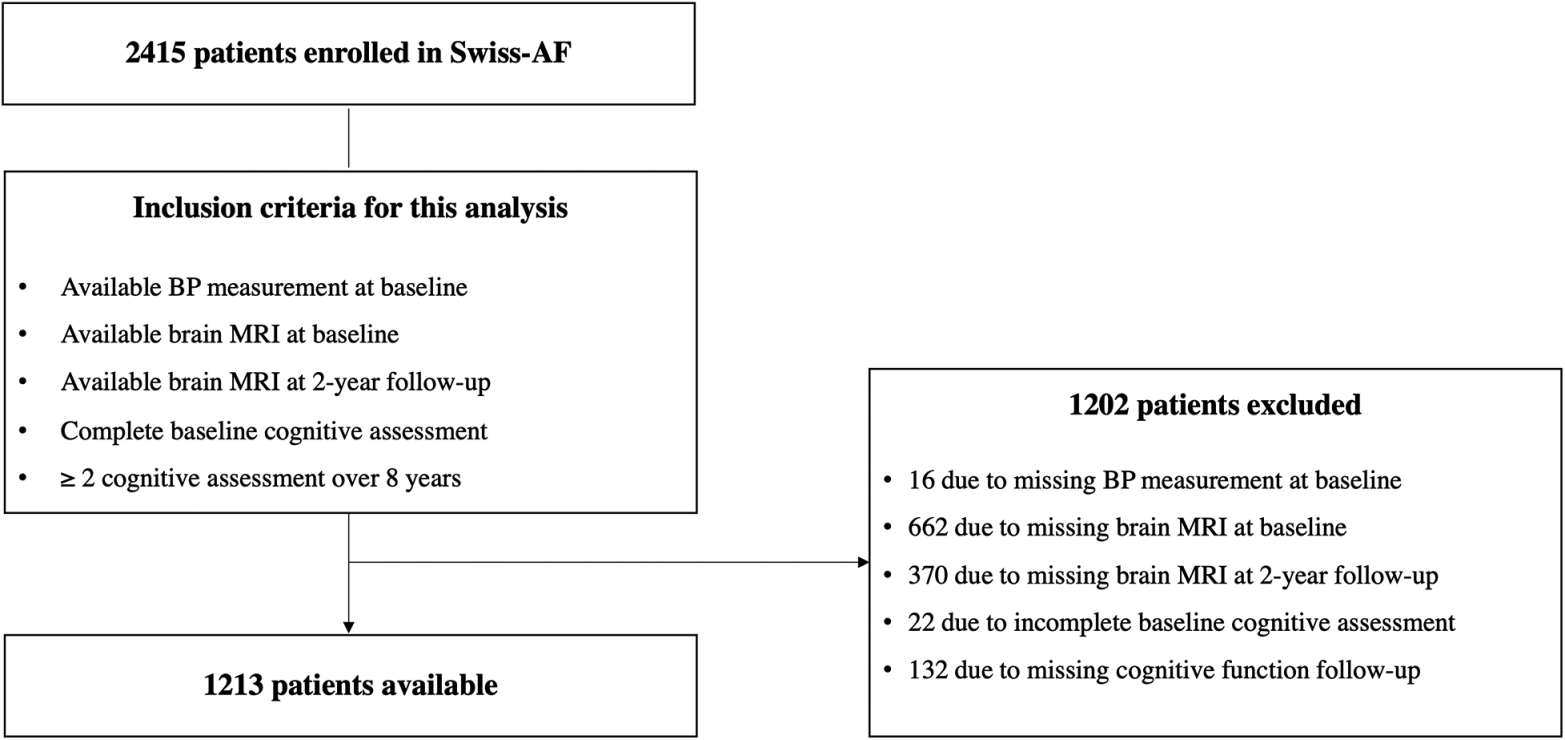
Flow chart patient enrollment. AF, atrial fibrillation; BP, blood pressure; MRI, magnetic resonance imaging.

The patient selection process is presented in Figure 1. Of the 2,415 patients enrolled in the Swiss-AF study, 16 patients were excluded due to missing BP measurement at baseline. Additionally, we excluded 662 patients with missing brain MRI at baseline (mainly due to a cardiac device or claustrophobia), and 370 patients with a missing brain MRI after 2 years. Finally, we had to exclude 22 patients with incomplete baseline cognitive assessment, and 132 patients with no follow-up assessment of cognitive functions, resulting in 1,213 participants for the current analysis.

### 2.2 Blood pressure measurement, blood pressure categories and other study variables

At baseline and during yearly follow-up visits, we collected detailed information about patient characteristics, educational status, medication, and medical history. History of hypertension was self-reported and confirmed through medical reports. Study patients had to complete standardized case report forms, containing questions about eating habits, physical activity, and smoking status. Regular physical activity was defined as patients performing sports at least once a week. Additionally, the geriatric depression scale (GDS) (19) was administered. Weight and height were measured, and the body mass index (BMI) was calculated by dividing weight in kilograms by height in meters squared. We recorded a resting 12-lead surface electrocardiogram (ECG) for 5 min and the rhythm was determined by board-certified cardiologists. At baseline we took blood samples, and we measured the levels of total cholesterol, and low-density lipoprotein-cholesterol (LDL-C). We defined hypercholesterolemia as either a total cholesterol level ≥200 mg/dl (5.2 mmol/dl) and/or LDL-C ≥ 190 mg/dl (4.9 mmol/dl).

We measured BP with validated devices three times in sitting or supine position after a resting period of 5 min. We then calculated the mean of the three consecutive BP measurements. For these analyses, we categorized the BP values into BP categories, according to the current guidelines of the European Society of Hypertension (20) (Supplementary Table S1).

### 2.3 Brain magnetic resonance imaging

A standardized brain MRI was conducted at baseline and after 2 years of follow-up. Every participating study center performed pre-specified, standardized brain MRI protocols with either 1.5 or 3 Tesla scanners and without contrast agents. The images were first locally evaluated for incidental findings, and then centrally analyzed at the Medical Image Analyses Center (MIAC AG) in Basel, Switzerland. Blinded expert raters and board-certified neuroradiologists performed standardized analyses and ratings.

We performed fluid attenuated inversion recovery (FLAIR) and susceptibility-weighted images (SWI), as well as T2*-weighted imaging. Non-cortical infarcts were defined as lesions that do not involve the cortex and show hyperintensity on axial section on FLAIR sequences. If the lesions presented a diameter of ≤20 mm, they were classified as small non-cortical infarcts (SNCI), otherwise as large non-cortical infarcts (LNCI) (21). Lesions that involve the cortex and typically showing hyperintensity in FLAIR were defined as cortical infarcts. We combined large non-cortical infarcts and any cortical infarct into one category (LNCCI). Ischemic lesions were defined as either LNCCI and/or SNCI. We defined WML as hyperintense lesions in T2*-weighted and FLAIR involving the periventricular or deep white matter. We also described the presence of Mb as nodular lesions, with strong hypointense signal on T2*-weighted or SWI. A composite endpoint for microvascular damage was defined including the presence of WML, Mb or SNCI. New lesions were defined as either lesions that appeared between the baseline and the 2-year follow-up or pre-existing lesions from the baseline that demonstrated an increase in volume after 2 years.

### 2.4 Neurocognitive testing

Neurocognitive testing was performed on a yearly basis using the Montreal Cognitive Assessment (MoCA), the Semantic Fluency Test (SFT), and the Digit Symbol Substitution Test (DSST). The complete test battery was done at in-person visits. During phone visits, only the SFT was used. In brief, the MoCA test is a screening instrument for mild cognitive dysfunction and aims to assess the global cognitive function, including tests for executive functions, visuoconstructional skills, attention, and memory (22). The maximum and best score is 30 points, the worst 0 points. Patients reporting an education of <12 years receive an additional point if they reach <30 points. The SFT is a brief test that assesses verbal ability and executive control (23). During the test, patients are given 60 s to list as many words as possible belonging to a specific category, such as animals (23). The DSST is a widely used cognitive test that requires patients to match symbols to numbers within a 120-second timeframe, with a maximum achievable score of 135 points (24). Motor speed, attention, and visuoperceptual functions are crucial factors that significantly influence performance in this task (24). Our main outcome was cognitive decline defined as a change of more than −1 standard deviation (SD) of the age-education standardized baseline population of the MoCA test, SFT and DSST, compared with individual baseline levels (25, 26). A drop of 1 SD in the standardized scores is equivalent to 3 MoCA points, 5 SFT score units, and 12 DSST score units.

### 2.5 Statistical analysis

Continuous variables are presented as mean (±SD) or median [interquartile range (IQR)] and categorical variables as numbers (percentage). Continuous systolic BP (SBP), continuous diastolic BP (DBP), and BP categories were considered as the main predictor variables. New brain lesions (occurrence and volume) and cognitive function were stratified by BP categories and were compared using the appropriate test (Chi-square test or Kruskal-Wallis-Test).

#### 2.5.1 Blood pressure and brain lesions

To assess the association of continuous SBP, continuous DBP and BP categories with presence of new brain lesions, multivariable adjusted logistic regression analyses were performed using, in turn, new ischemic infarcts, WML, Mb and a composite (SNCI, Mb and WML) as the outcome variables. Linear regression analyses were done to assess the association of BP and BP categories with new lesions’ volume. To improve the normality of the distribution, the volume of the brain lesions was log-transformed. We calculated p for linear and quadratic trends, and we performed interaction analysis to examine potential effect modification of AF type (paroxysmal vs. non-paroxysmal) and baseline heart rhythm (AF and atrial flutter vs. sinus rhythm) on the association of BP with the incidence of new ischemic lesions and new WML.

All regression models were adjusted for potential confounders, pre-chosen based on expert knowledge. The first model was adjusted for sex and age. A second model was additionally adjusted for educational status, BMI, smoking status, previous stroke, or transient ischemic attack (TIA), history of diabetes, history of heart failure, history of coronary heart disease, AF type, oral anticoagulation, antithrombotic treatment, and antihypertensive treatment.

#### 2.5.2 Blood pressure and cognitive function

To assess the associations of continuous BP and BP categories with cognitive decline over a time period of up to 8 years, we performed multivariable adjusted Cox proportional hazard regression analyses. We used age-education adjusted standardized scores for all cognitive outcomes of interest. To determine person-years of follow-up, we measured the time from baseline to either cognitive decline, the last visit, or the last visit preceding study termination due to drop-outs, death, or loss to follow-up.

We adjusted the first model for sex, age, and educational status. The second model was additionally adjusted for GDS, BMI, smoking status, previous stroke or TIA, diabetes, heart failure, coronary heart disease, AF type, oral anticoagulation, antithrombotic treatment, and antihypertensive treatment.

Since loss of patients due to death may generate an attrition bias on the estimates, we conducted a sensitivity analysis by repeating the Cox regression analyses without patients who died during the course of the study. As additional sensitivity analysis, we reanalyzed the association of BP with cognitive decline, by using our dataset together with initially excluded patients due to missing MRI data (*n* = 2,124). Another sensitivity analysis was performed by adding regular physical activity and hypercholesterolemia as covariates (Model 3). Furthermore, to investigate the association of baseline BP with cognitive function scores as continuous variable, we performed mixed effects linear models. Time since the first measurement (random slope) and patient number nested within center (random intercepts) were added to both models. As fixed effects we included the same adjusting variables as described for models 1 and 2, alongside interaction terms between time and BP.

We considered a two-sided *p*-value <0.05 as statistically significant; no correction was done for multiple testing. All statistical analyses were performed using R version 4.2.2 (Foundation for Statistical Computing, Vienna, Austria).

## 3 Results

### 3.1 Baseline characteristics

Baseline characteristics are presented in Table 1 and a comparison of the final patient population with patients without complete MRI data is shown in Supplementary Table S2. Mean (SD) age of the 1,213 included patients was 71.4 (±8.4) years and 897 (73.9%) patients were male. Mean systolic/diastolic BP was 135/79 (±18/12) mmHg and mean CHA_2_DS_2_-VASc score was 3.1 (±1.7) points. Most patients were in sinus rhythm at the baseline visit (*n* = 673, 55.7%) and 46.7% (*n* = 567) had paroxysmal AF. A history of hypertension was reported by 821 (67.7%) patients, 792 (65.3%) were already on antihypertensive medication, and 47.1% required ≥3 antihypertensive drugs. Overall, 89.5% (*n* = 1,086) were prescribed oral anticoagulation, while 17.2% (*n* = 208) received antiplatelet therapy.

**Table 1:** Baseline characteristics.

	Study population
	*n* = 1,213
Age, years	71.4 (±8.4)
Sex, male, %	897 (73.9)
Body mass index, kg/m^2^	27.7 (±4.7)
Education, years	13 (±3)
Geriatric depression scale, points	1 [0, 2]
Active smoking, %	84 (6.9)
Regular physical activity, %	634 (52.3)
Heart rate, bpm	66 [57, 77]
Systolic blood pressure, mmHg	135 (±18)
Diastolic blood pressure, mmHg	79 (±12)
Blood pressure categories, %	
Optimal	211 (17.4)
Normal	249 (20.5)
High normal	265 (21.8)
Hypertension grade 1	348 (28.7)
Hypertension grade 2 and 3	140 (11.5)
Atrial fibrillation type, %	
Paroxysmal	567 (46.7)
Non-paroxysmal	646 (53.3)
Heart rhythm at the baseline visit, %	
Sinus rhythm	673 (55.7)
Atrial fibrillation	408 (33.7)
Atrial flutter	43 (3.6)
Other	85 (7.0)
CHA_2_DS_2_-VASc score, points	3.1 (±1.7)
History of arterial hypertension, %	821 (67.7)
Antihypertensive treatment, %	792 (65.3)^a^
Number of antihypertensive drugs, %	
1	142 (17.9)
2	277 (35.0)
≥3	373 (47.1)
History of heart failure, %	228 (18.8)
History of diabetes mellitus, %	173 (14.3)
History of stroke or TIA, %	232 (19.1)
History of coronary heart disease, %	312 (25.7)
History of renal failure, %	176 (14.5)
History of systemic embolism, %	52 (4.3)
History of peripheral artery disease, %	64 (5.3)
History of OSAS, %	157 (12.9)
Hypercholesterolemia, %	325 (28.4)
Oral anticoagulation, %	1,086 (89.5)
Antiplatelet therapy, %	208 (17.2)
Brain magnetic resonance imaging	
Ischemic lesions, %	428 (35.4)
LNCCI, %	252 (20.9)
SNCI, %	250 (20.7)
Volume, mm^3^	2,163 [594, 9,596]
White matter lesions, %	1,195 (98.9)
Volume, mm^3^	3,336 [1,231, 8,599]
Microbleeds, %	238 (20.3)
Cognitive testing	
MoCA score, points	26 [24, 28]
Semantic fluency test, points	20 [16, 23]
Digit symbol substitution test, points	47 [37, 56]

Data are presented as *n* (%), mean (±standard deviation), or median [interquartile range]. BP, blood pressure; MoCA, Montreal cognitive assessment; LNCCI, large non-cortical and cortical infarcts; OSAS, obstructive sleep apnea syndrome; SNCI, small non-cortical infarcts; TIA, transient ischemic attack; and WML, white matter lesions.

Optimal BP indicates systolic BP <120 and diastolic BP <80 mmHg; Normal BP, systolic BP 120–129 mmHg and/or diastolic BP 80–84 mmHg; High normal BP, systolic BP 130–139 mmHg and/or diastolic BP 85–89 mmHg; Hypertension grade 1, systolic BP 140–159 mmHg and/or diastolic BP 90–99 mmHg; Hypertension grade 2 and 3, systolic BP ≥160 and/or diastolic BP ≥100 mmHg.

Missings: Geriatric depression scale *n* = 1; Heart rhythm at the baseline visit *n* = 4; Hypercholesterolemia *n* = 68; Antiplatelet therapy *n* = 1; Ischemic lesions *n* = 5; White matter lesions *n* = 5; Microbleeds *n* = 38.

^a^ Of the 821 patients with hypertension, 792 (96.5%) were on antihypertensive treatment.

When considering the measured BP at the baseline visit, 211 patients (17.4%) showed optimal BP (SBP <120 and DBP <80 mmHg), 249 (20.5%) normal BP (SBP 120–129 and/or DBP 80–84 mmHg), and 265 (21.8%) high normal BP levels (SBP 130–139 and/or DBP 85–89 mmHg). Most patients (*n* = 348, 28.7%) had hypertension grade 1 (SBP 140–159 and/or DBP 90–99 mmHg), and 140 (11.5%) patients had hypertension grade 2 or 3 (SBP ≥160 and/or DBP ≥100 mmHg).

At baseline, ischemic brain lesions were present in 428 (35.4%) patients, 1,195 (98.9%) patients had WML, and Mb were present in 238 (20.3%) patients. The overall median MoCA score was 26 [24, 28] points, the median SFT was 20 [16, 23] points, and the median DSST 47 [37, 56] points. The cognitive test scores did not differ among different BP categories (Supplementary Table S3).

### 3.2 Blood pressure and new brain lesions

**Figure 2:**
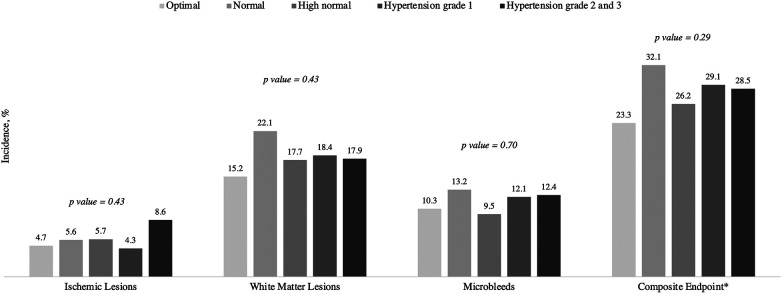
Incidence of new brain lesions by blood pressure categories. Data are %. *P*-value was calculated using the *χ*2 tests or Kruskal-Wallis Tests, as appropriate. *Composite endpoint for microvascular damage of white matter lesions, microbleeds and small non-cortical infarcts. BP, blood pressure. Optimal BP indicates systolic BP <120 and diastolic BP <80 mmHg; Normal BP, systolic BP 120–129 mmHg and/or diastolic BP 80–84 mmHg; High normal BP, systolic BP 130-139 mmHg and/or diastolic BP 85-89 mmHg; Hypertension grade 1, systolic BP 140-159 mmHg and/or diastolic BP 90-99 mmHg; Hypertension grade 2 and 3, systolic BP ≥160 and/or diastolic BP ≥100 mmHg.

The overall incidence of new ischemic lesions after 2-years follow-up was 5.4%, with a new median volume of 278 [116, 653] mm^3^. New or enlarged WML were present in 223 (18.4%) patients with a median volume of 108 [45, 284] mm^3^. Mb and the composite outcome of microvascular damage were present in 11.5% and 28% of patients, respectively (Table 2, Figure 2). There were no significant differences in the incidence or volume of brain lesions across the various BP categories.

**Table 2:** Incidence and volume of new brain lesions on brain magnetic resonance imaging after 2 years of follow-up, overall and in blood pressure categories.

		Ischemic lesions		White matter lesions		Microbleeds	Composite endpoint^a^
	*n* (%)	Incidence	Volume, mm^3^	Incidence	Volume, mm^3^	Incidence	Incidence
Overall	1,213	66 (5.4)	278 [116, 653]	223 (18.4)	108 [45, 284]	135 (11.5)	332 (28.0)
Blood pressure categories							
Optimal	211 (17.4)	10 (4.7)	534 [203, 799]	32 (15.2)	138 [65, 315]	21 (10.3)	48 (23.3)
Normal	249 (20.5)	14 (5.6)	477 [109, 993]	55 (22.1)	105 [41, 590]	32 (13.2)	79 (32.1)
High normal	265 (21.8)	15 (5.7)	315 [134, 608]	47 (17.7)	75 [42, 179]	24 (9.5)	67 (26.2)
Hypertension grade 1	348 (28.7)	15 (4.3)	207 [48, 269]	64 (18.4)	95 [35, 335]	41 (12.1)	99 (29.1)
Hypertension grade 2 and 3	140 (11.5)	12 (8.6)	332 [112, 494]	25 (17.9)	159 [90, 249]	17 (12.4)	39 (28.5)
*P*-value		0.43	0.22	0.43	0.21	0.70	0.29

Data are numbers (%) or median [interquartile range]. BP, blood pressure.

Groups were compared using *χ*2 tests or Kruskal-Wallis Tests, as appropriate.

Optimal BP indicates systolic BP <120 and diastolic BP <80 mmHg; Normal BP, systolic BP 120–129 mmHg and/or diastolic BP 80–84 mmHg; High normal BP, systolic BP 130–139 mmHg and/or diastolic BP 85–89 mmHg; Hypertension grade 1, systolic BP 140–159 mmHg and/or diastolic BP 90–99 mmHg; Hypertension grade 2 and 3, systolic BP ≥160 and/or diastolic BP ≥100 mmHg.

Valid patients for microbleeds *n* = 1,174; Valid patients for composite endpoint *n* = 1,185.

^a^ Composite endpoint for microvascular damage of white matter lesions, microbleeds and small non-cortical infarcts.

**Figure 3:**
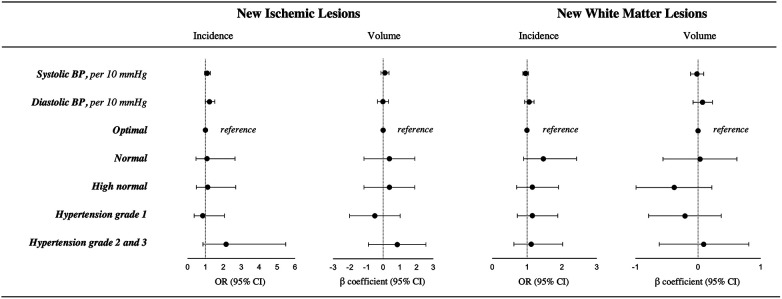
Results of the multivariable adjusted logistic and linear regression model of the association between blood pressure and the incidence and volume of new ischemic infarcts and new white matter lesions. Models are adjusted for age, sex, education, body mass index, smoking status, previous stroke or transient ischemic attack, history of diabetes, history of heart failure, history of coronary heart disease, atrial fibrillation type, oral anticoagulation, antithrombotic treatment, and antihypertensive treatment. BP, blood pressure; CI, confidence interval; and OR, odds ratio. Optimal BP indicates systolic BP <120 and diastolic BP <80 mmHg; Normal BP, systolic BP 120–129 mmHg and/or diastolic BP 80–84 mmHg; High normal BP, systolic BP 130–139 mmHg and/or diastolic BP 85–89 mmHg; Hypertension grade 1, systolic BP 140-159 mmHg and/or diastolic BP 90-99 mmHg; Hypertension grade 2 and 3, systolic BP ≥160 and/or diastolic BP ≥100 mmHg. Valid *n* for incidence for all patients = 1,213. Valid *n* for volume of new ischemic lesions = 66. Valid *n* for volume of new white matter lesions = 223.

The results of the association analyses between BP and different types of brain lesions are presented in Figure 3, Supplementary Tables S4, and S5. After adjusting for a comprehensive set of variables, the incidence and volume of ischemic lesions were not associated with BP categories. Per 10 mmHg increase in SBP and DBP the adjusted odds ratio (OR) [95% confidence interval (CI)] for new ischemic lesions were 1.10 (0.95–1.27; *p* = 0.21) and 1.23 (0.99–1.52, *p* = 0.06), respectively. The adjusted *β*-coefficients (95% CI) for the association of continuous SBP and DBP with the volume of ischemic lesions were 0.11 (−0.13 to 0.35, *p* = 0.37), and −0.01 (−0.34 to 0.32, *p* = 0.94).

When investigating the association of BP with the occurrence of new WML, there was no trend across BP categories (*p* for linear trend = 0.99, *p* for quadratic trend = 0.43). Per 10 mmHg increase in SBP and DBP the adjusted OR for incidence of WML were 0.96 (0.88 to 1.04; *p* = 0.32) and 1.06 (0.93 to 1.20, *p* = 0.40). Similarly, there was no association between BP and the volume of new WML. The results of the association of BP with new Mb and the composite outcome are presented in Supplementary Table S5 and Figure S1. None of the associations were significant.

Subgroup and interaction analyses of the association between SBP, ischemic lesions and WML are presented in Supplementary Table S6. AF type (paroxysmal vs. non-paroxysmal) and heart rhythm (AF and atrial flutter vs. sinus rhythm) did not show any interaction effect on the association between continuous SBP and the incidence of new ischemic lesions or WML.

### 3.3 Blood pressure and cognitive decline

The results of the association between BP and cognitive decline are presented in Table 3. The median follow-up time for the MoCA or DSST was 4 years and 6 years for the SFT. The incidence rate per 100 person years for cognitive decline was 2.50 when using the MoCA score, 6.20 for the SFT, and 2.31 for the DSST. Per 10 mmHg increase in SBP, the adjusted hazard ratio (HR) for cognitive decline was 0.94 (0.85–1.04, *p* = 0.23) using the MoCA score, 0.94 (0.89–1.00, *p* = 0.06) using the SFT, and 0.94 (0.84–1.05, *p* = 0.27) when using the DSST. Across increasing BP categories, we could not find any significant association between BP categories and cognitive decline using the MoCA Score (*p* for linear trend = 0.38, *p* for quadratic trend = 0.57) or using the DSST (*p* for linear trend = 0.52, *p* for quadratic trend = 0.99). However, using the SFT and considering optimal BP as reference, adjusted HR (95% CI) for cognitive impairment was 0.75 (0.55–1.03, *p* = 0.07) for normal BP, 0.63 (0.46–0.87, *p* = 0.005) for high normal BP, 0.57 (0.42–0.77, *p* < 0.001) for hypertension grade 1, and 0.72 (0.49–1.05, *p* = 0.09) for hypertension grade 2 and 3; with *p* for linear trend = 0.025 and *p* for quadratic trend = 0.020. The results of our sensitivity analysis excluding patients who died during the study period were consistent with the results of our main analysis (Supplementary Table S7).

**Table 3:** Association of blood pressure and hypertension categories with new cognitive decline by using Cox regression analysis.

	Number of events	Patient-years	Incidence rate per 100 patient-years	Cox regression Model 1 HR (95% CI), *p-*value	Cox regression Model 2 HR (95% CI), *p-*value
MoCA	126	5,042	2.50		
Systolic BP, per 10 mmHg				0.94 (0.85 to 1.03), *p* = 0.19	0.94 (0.85 to 1.04), *p* = 0.23
Diastolic BP, per 10 mmHg				0.87 (0.75 to 1.02), *p* = 0.08	0.87 (0.74 to 1.02), *p* = 0.08
Blood pressure categories					
Optimal	21	858	2.45	*reference*	*reference*
Normal	25	1,047	2.39	0.86 (0.48 to 1.55), *p* = 0.62	0.85 (0.47 to 1.53), *p* = 0.59
High normal	36	1,098	3.28	1.19 (0.69 to 2.04), *p* = 0.53	1.19 (0.69 to 2.07), *p* = 0.53
Hypertension grade 1	31	1,461	2.12	0.74 (0.43 to 1.29), *p* = 0.29	0.76 (0.43 to 1.34), *p* = 0.34
Hypertension grade 2 and 3	13	578	2.25	0.77 (0.39 to 1.54), *p* = 0.46	0.75 (0.37 to 1.53), *p* = 0.43
P for linear trend				0.37	0.38
P for quadratic trend				0.61	0.57
SFT	370	5,966	6.20		
Systolic BP, per 10 mmHg				0.95 (0.89 to 1.00), *p* = 0.07	0.94 (0.89 to 1.00), *p* = 0.06
Diastolic BP, per 10 mmHg				0.95 (0.87 to 1.04), *p* = 0.27	0.95 (0.87 to 1.04), *p* = 0.30
Blood pressure categories					
Optimal	84	995	8.44	*reference*	*reference*
Normal	81	1,214	6.67	0.78 (0.57 to 1.06), *p* = 0.11	0.75 (0.55 to 1.03), *p* = 0.07
High normal	73	1,328	5.50	0.64 (0.47 to 0.88), *p* = 0.005	0.63 (0.46 to 0.87), *p* = 0.005
Hypertension grade 1	89	1,771	5.03	0.58 (0.43 to 0.78), *p* < 0.001	0.57 (0.42 to 0.77), *p* < 0.001
Hypertension grade 2 and 3	43	658	6.53	0.76 (0.52 to 1.10), *p* = 0.15	0.72 (0.49 to 1.05), *p* = 0.09
P for linear trend				0.039	0.025
P for quadratic trend				0.015	0.020
DSST	116	5,019	2.31		
Systolic BP, per 10 mmHg				0.95 (0.85 to 1.05), *p* = 0.30	0.94 (0.84 to 1.05), *p* = 0.27
Diastolic BP, per 10 mmHg				0.95 (0.81 to 1.12), *p* = 0.56	0.97 (0.83 to 1.14), *p* = 0.73
Blood pressure categories					
Optimal	20	849	2.36	*reference*	*reference*
Normal	28	1,021	2.74	1.16 (0.66 to 2.07), *p* = 0.60	1.16 (0.65 to 2.06), *p* = 0.62
High normal	27	1,122	2.41	1.00 (0.56 to 1.79), *p* = 0.998	1.00 (0.56 to 1.79), *p* = 0.999
Hypertension grade 1	28	1,456	1.92	0.79 (0.44 to 1.40), *p* = 0.42	0.78 (0.43 to 1.40), *p* = 0.40
Hypertension grade 2 and 3	13	571	2.28	0.96 (0.48 to 1.94), *p* = 0.92	0.95 (0.47 to 1.94), *p* = 0.89
P for linear trend				0.54	0.52
P for quadratic trend				0.99	0.99

Decline of cognitive function was defined as a decrease of one age-education normalized standard deviation from each patient's test baseline value. Model 1 was adjusted for age, sex, and education; *n* = 1,213.

Model 2 was additionally adjusted for geriatric depression score, body mass index, smoking status, previous stroke or transient ischemic attack, history of diabetes, history of heart failure, history of coronary heart disease, atrial fibrillation type, oral anticoagulation, antithrombotic treatment, and antihypertensive treatment; *n* = 1,212.

BP, blood pressure; CI, confidence interval; DSST, digit symbol substitution test; HR, hazard ratio; MoCA, Montreal Cognitive Assessment; and SFT, semantic fluency test.

Optimal BP indicates systolic BP <120 and diastolic BP <80 mmHg; Normal BP, systolic BP 120–129 mmHg and/or diastolic BP 80–84 mmHg; High normal BP, systolic BP 130–139 mmHg and/or diastolic BP 85–89 mmHg; Hypertension grade 1, systolic BP 140–159 mmHg and/or diastolic BP 90–99 mmHg; Hypertension grade 2 and 3, systolic BP ≥160 and/or diastolic BP ≥100 mmHg.

A sensitivity analysis of 2,124 patients, also including patients without MRI data, showed significant associations between continuous DBP and cognitive decline by using the MoCa score, as well as between continuous BP and BP categories with cognitive decline by using the SFT (Supplementary Table S8). The sensitivity analysis considering physical activity and hypercholesterolemia as possible confounders (Supplementary Table S9) showed consistent results to our main analysis. Results of the mixed effects linear models did not show any significant interactions between BP and time, thus do not support a change in cognition over time to depend on BP (Supplementary Table S10).

## 4 Discussion

In this large cohort of AF patients, we investigated the associations of BP with new brain lesions after 2 years and cognitive decline over a time period of up to 8 years (median 6 years). The main findings are the following: first, nearly 68% of our patients had a prior history of hypertension at baseline. Second, after 2 years, there was no substantial association observed between BP and the occurrence and extent of new brain lesions on brain MRI. Third, BP was not consistently associated with cognitive decline during a median follow-up of 6 years.

Although we had observed a significant association between BP and brain lesions in a cross-sectional study (16), we found no strong evidence of an association between BP and new brain lesions after a mid-term follow-up of 2 years. Several factors might account for these findings. First, the limited number of ischemic and hemorrhagic events, potentially due to the high percentage of patients on oral anticoagulation and antihypertensive treatment (27–29). Second, considering the number of patients with pre-existing brain lesions at baseline, it is plausible that the observation period of 2 years might be insufficient to see relevant changes in the occurrence and volume of new lesions, despite high BP levels in some patients. Furthermore, the possible dynamic nature of brain lesions and the sensitivity of brain MRI warrants consideration, as it may have led to failure to detect brain lesions and subsequent underestimation of new lesions (30, 31). A longer term analysis may be able to uncover an association between BP levels and brain lesions, considering the potential rise in event rates over time. Moreover, it could enhance our understanding of how the impact of BP levels evolves over time (midlife vs. late-life) in patients with AF.

In our analysis, the association between BP and cognitive decline was inconsistent and depended on the type of cognitive test examined. We found some support for a possible association of cognitive decline in SFT with BP, but no evidence for this in the other two cognitive tests. The MoCa score is a relevant clinical screening tool to identify mild dementia and mild cognitive impairment (32). It includes the evaluation of executive function, visuospatial abilities, short-term and working memory, naming, language, attention, concentration, verbal abstraction, and orientation (22, 32). As the MoCa score covers various cognitive functions, it is a more global cognitive assessment tool. Thus, it might be possible that strengths in some functions can compensate weaknesses in others. This could result in a reduced event rate (compared to more specific cognitive assessments) and could potentially explain the lack of significant associations observed in our analysis.

The DSST has also been shown to measure various cognitive operations [such as attention, visuoperceptual functions, associative learning, working memory and motor speed (24)]. Though it is a useful and sensitive test to monitor cognitive dysfunction. However, it has a low specificity to identify which specific cognitive domain is affected (24) and a slight impairment in a specific cognitive function might be compensated by strengths in others. Such a compensation may be less likely in the SFT, as it assesses fewer cognitive functions [namely semantic memory and executive function (33, 34)]. However, considering the potential impact of the number of events and the larger sample size for the SFT on statistical power, it is not clear whether there is indeed a specific influence of BP on the SFT, or whether the differences compared to the other neurocognitive tests are real or a chance-finding.

Though the statistical significance level were not reached, the Cox regression analysis showed lower HRs in patients with higher BP (compared to patients with optimal BP), suggesting that increased BP levels may play an active role in the development of cognitive impairment. Recent findings in the general population are comparable with our data, possibly underlining the importance of maintaining an adequate level of DBP in patients affected with AF (35). Current literature suggests that cerebral perfusion might play a key role in cognitive function (36). Studies have shown a link between higher cerebral perfusion and better cognitive performance (37), as well as cerebral hypoperfusion with cognitive impairment (38). A history of coronary heart disease was reported by 25.7% of the patients, and 5.3% had peripheral artery disease. In our cohort of patients mainly over 65 years, arterial stiffness and vascular calcification was not systematically assessed, but may be present in a majority of patients. Considering that these conditions lead to impaired brain perfusion, the higher BP levels which may have contributed to vascular damages in midlife, may permit a sufficient and better perfusion, leading to better cognitive performance later (39, 40). A further possible explanation could be hidden behind the increased beat-to-beat variation in AF, which leads to intermittent cerebral hypoperfusion (41). The slightly elevated BP levels may permit the counteraction of the reduction in cerebral blood flow and maintain the perfusion more stable.

The findings of the cognitive tests could be influenced by the specific brain regions involved in each task. The SFT relies on areas like the temporal and frontal regions (particularly the prefrontal cortex), which are crucial for executive functions (42, 43). It is conceivable that patients with AF who are at higher risk for brain lesions, embolic events, and comorbidities (10, 44, 45), might benefit from slightly elevated BP levels, which improve blood flow in these regions. Advanced brain MRI protocols, which include the estimation of brain perfusion, could be helpful to better investigate this association in patients with AF.

Furthermore, several studies have underlined the importance of age, which may strongly influence the association between BP and cognitive function (39, 40, 46). BP levels and hypertension might have their greatest and negative influence on cognitive function during midlife (48–67 years) (47). Considering that our study population had a mean age of 72 years, and was mainly aged over 65 years, we may have missed the patient categories in which potential effects on neurocognitive function later in life might be significant.

For our analysis, we mainly focused on the BP measured at baseline, regardless of whether patients had diagnosed hypertension or if they were on antihypertensive therapy. However, the time point of the hypertension diagnosis, the persistence and a sufficient duration of high BP levels also play an important role in order to detect significant influence on cognitive function and cognitive decline (40).

The SPRINT (48) and the SPRINT-MIND (49) study provided important results regarding intensive BP control (target SBP of ≤120 mmHg) and the association with cardiovascular events, dementia and mild cognitive impairment (MCI). Compared to our analysis, the SPRINT-MIND study did not find any significant effects on incidence of dementia. However, they found benefits regarding the progression of WML and a lower risk of MCI. A post-hoc analysis in patients with AF showed a positive association of intensive BP treatment on the risk of dementia but no significant associations with MCI or probable dementia (50). In our study population, nearly 97% of patients with history of hypertension were on antihypertensive treatment. The question concerning the potential benefit of antihypertensive treatment and the best therapeutic goal for elderly AF patients with a high burden of cardiovascular disease thus remains unanswered. Considering the high prevalence of AF and the increasing rate of dementia, further study of this topic may be of relevant public interest.

### 4.1 Strengths and limitations

The strengths of our study include the detailed information concerning patient characteristics and the large sample of AF patients with brain MRI data. We performed systematic and standardized brain MRI and cognitive assessment, which increases the reliability and comparability of the results over up to 8 years and across 14 centers in the three main language regions in Switzerland. However, certain limitations should be considered when interpreting the results. First, the generalizability of our results to other patient populations is unclear as we mainly included patients of European origin. Similarly, our findings are limited to patients who were able to undergo brain MRI. Second, the follow-up duration of 2 years for the MRI analysis and up to 8 years for the cognitive decline analysis might be too short and may represent a limitation of the analysis. Third, due to the limited number of patients with ischemic lesions after 2 years, we could not analyze LNCCI and SNCI separately. We combined them as ischemic infarcts instead. The merging of the two categories could have caused some noise, as these entities likely have different pathophysiological backgrounds (11, 21). The limited event rate may also affect the statistical power of our analyses. Finally, Swiss-AF is an observational study. Although potential confounders were included in our models as adjusting variables, residual confounding may have influenced our results.

## 5 Conclusion

In a well-characterized cohort of AF patients, we did not find compelling evidence for an association between BP and incidence or volume of new brain lesions after a follow-up of 2 years. The results of certain BP categories and a specific cognitive test indicate a potential risk reduction for cognitive decline; however, BP was not consistently associated with cognitive decline during a median follow-up of 6 years.

## Data Availability

The data analyzed in this study is subject to the following licenses/restrictions: the datasets presented in this article are not publicly available due to restrictions by the Ethics Committee. Requests to access these datasets should be directed to Michael Kühne, michael.kuehne@usb.ch.
