# Supplementary material for: Blood pressure, brain lesions and cognitive decline in patients with atrial fibrillation
Source: Front Cardiovasc Med. 2024 Sep 3;11:1449506. doi: 10.3389/fcvm.2024.1449506 (PMC11417621; doi:10.3389/fcvm.2024.1449506)
Supplement: Supplementary file 1 [file Datasheet1.pdf]

BLOOD PRESSURE, BRAIN LESIONS AND COGNITIVE DECLINE IN PATIENTS WITH ATRIAL FIBRILLATION

BASELINE

- 1213 patients with AF
- Mean age 71 years
- 74% male

- Mean BP 135/79 mmHg
- History of hypertension 68%
- Antihypertensive therapy 97%

- Ischemic lesions 35%
- White Matter Lesions 99%
- Microbleeds 20%

Standardized brain MRI
At baseline and after 2 years

Standardized cognitive testing
Yearly

NEW BRAIN LESIONS

- Follow-up time: 2 years

- New Ischemic lesions 5.4%
- New White Matter Lesions 18.4%
- New Microbleeds 11.5%

No evidence for an association between BP and new brain lesions after 2 years

COGNITIVE DECLINE

- Median Follow-up time: 6 years

BP was not consistently associated with cognitive decline

AF	=	Atrial Fibrillation
BP	=	Blood Pressure
MRI	=	Magnetic Resonance Imaging
