# Supplementary material for: Blood pressure, brain lesions and cognitive decline in patients with atrial fibrillation
Source: Front Cardiovasc Med. 2024 Sep 3;11:1449506. doi: 10.3389/fcvm.2024.1449506 (PMC11417621; doi:10.3389/fcvm.2024.1449506)
Supplement: Supplementary file 2 [file Datasheet2.docx]

Supplementary Material

**Blood pressure, brain lesions and cognitive decline in patients with atrial fibrillation**

Désirée Carmine, Stefanie Aeschbacher, Michael Coslovsky, Elisa Hennings, Rebecca E. Paladini, Raffaele Peter, Melanie Burger, Tobias Reichlin, Nicolas Rodondi, Andreas S. Müller, Peter Ammann, Giulio Conte, Angelo Auricchio, Giorgio Moschovitis, Julia B. Bardoczi, Annina Stauber, Maria Luisa De Perna, Christine S. Zuern, Tim Sinnecker, Patrick Badertscher, Christian Sticherling, Leo H. Bonati, David Conen, Philipp Krisai, Stefan Osswald, Michael Kühne, for the Swiss-AF Investigators

1. **Supplementary Data**
   1. **Swiss-AF Investigators**
2. **Supplementary Tables**

**Table S1: Blood pressure classification**

**Table S2: Comparison baseline characteristics of patients with and without**

**MRI at baseline and after 2 years**

**Table S3: Results of cognitive testing at baseline stratified by blood pressure**

**categories**

**Table S4: Association between blood pressure with new ischemic lesions and**

**new white matter lesions**

**Table S5: Association between blood pressure with new microbleeds and composite endpoint**

**Table S6: Interaction analysis for the association of blood pressure with new**

**ischemic lesions and new white matter lesions**

**Table S7: Sensitivity analysis: Association between blood pressure and**

**cognitive decline in patients without study termination**

**Table S8: Sensitivity analysis: Association between blood pressure and cognitive decline including patients without MRI data**

**Table S9: Sensitivity analysis: Association between blood pressure and cognitive decline additionally adjusted for regular physical activity**

**and hypercholesterolemia**

**Table S10: Sensitivity analysis: Mixed effects linear models for baseline blood pressure and cognitive function**

1. **Supplementary Figures**

**Figure S1: Logistic regression models for incidence of new microbleeds and**

**composite endpoint**

# Supplementary Data

## Swiss-AF Investigators

University Hospital Basel and Basel University: Stefanie Aeschbacher, Steffen Blum, Leo Bonati, Désirée Carmine, David Conen, Ludvig Dahlheim, Urs Fischer, Andreas Gasser, Corinne Girroy, Elisa Hennings, Philipp Krisai, Michael Kühne, Vincent Meier, Christine Meyer-Zürn, Pascal Meyre, Andreas U. Monsch, Christian Müller, Stefan Osswald, Rebecca E. Paladini, Raffaele Peter, Adrian Schweigler, Christian Sticherling, Gian Völlmin.

Principal Investigator: Stefan Osswald; Local Principal Investigator: Michael Kühne.

University Hospital Bern: Faculty: Drahomir Aujesky, Juerg Fuhrer, Laurent Roten, Simon Jung, Heinrich Mattle; Research fellows: Seraina Netzer, Luise Adam, Carole Elodie Aubert, Martin Feller, Axel Loewe, Elisavet Moutzouri, Claudio Schneider; Study nurses: Tanja Flückiger, Cindy Groen, Lukas Ehrsam, Sven Hellrigl, Alexandra Nuoffer, Damiana Rakovic, Nathalie Schwab, Rylana Wenger, Tu Hanh Zarrabi Saffari. Local Principal Investigators: Nicolas Rodondi, Tobias Reichlin.

Stadtspital Triemli Zurich: Roger Dillier, Michèle Deubelbeiss, Franz Eberli, Christine Franzini, Isabel Juchli, Claudia Liedtke, Samira Murugiah, Jacqueline Nadler, Thayze Obst, Jasmin Roth, Fiona Schlomowitsch, Xiaoye Schneider, Peter Sporns, Katrin Studerus, Noreen Tynan, Dominik Weishaupt. Local Principal Investigator: Andreas Müller.

Kantonspital Baden: Corinne Friedli, Silke Kuest, Karin Scheuch, Denise Hischier, Nicole Bonetti, Alexandra Grau, Jonas Villinger, Eva Laube, Philipp Baumgartner, Mark Filipovic, Marcel Frick, Giulia Montrasio, Stefanie Leuenberger, Franziska Rutz. Local Principal Investigator: Jürg-Hans Beer.

Cardiocentro Lugano: A Angelo Auricchio, Adriana Anesini, Cristina Camporini, Maria Luce Caputo, Rebecca Peronaci, Francois Regoli, Martina Ronchi. Local Principal Investigator: Giulio Conte.

Kantonsspital St. Gallen: Roman Brenner, David Altmann, Karin Fink, Michaela Gemperle. Local Principal Investigator: Peter Ammann.

Hôpital Cantonal Fribourg: Mathieu Firmann, Sandrine Foucras, Martine Rime. Local Principal Investigator: Michael Kühne.

Luzerner Kantonsspital: Benjamin Berte, Kathrin Bühler, Virgina Justi, Frauke Kellner-Weldon, Richard Kobza, Melanie Koch, Brigitta Mehmann, Sonja Meier, Myriam Roth, Andrea Ruckli-Kaeppeli, Ian Russi, Kai Schmidt, Mabelle Young. Local Principal Investigator: Michael Kühne.

Ente Ospedaliero Cantonale Lugano: Elia Rigamonti, Carlo Cereda, Alessandro Cianfoni, Maria Luisa De Perna, Jane Frangi-Kultalahti, Patrizia Assunta Mayer Melchiorre, Anica Pin, Tatiana Terrot, Luisa Vicari. Local Principal Investigator: Giorgio Moschovitis.

University Hospital Geneva: Georg Ehret, Hervé Gallet, Elise Guillermet, Francois Lazeyras, Karl-Olof Lovblad, Patrick Perret, Philippe Tavel, Cheryl Teres. Local Principal Investigator: Dipen Shah.

University Hospital Lausanne: Nathalie Lauriers, Marie Méan, Alessandra Pia Porretta, Sandrine Salzmann, Jürg Schläpfer. Local Principal Investigator: Michael Kühne.

Bürgerspital Solothurn: Andrea Grêt, Jan Novak, Sandra Vitelli. Local Principal Investigator: Frank-Peter Stephan.

Ente Ospedaliero Cantonale Bellinzona: Jane Frangi-Kultalahti, Augusto Gallino, Luisa Vicari. Local Principal Investigator: Marcello Di Valentino.

University of Zurich/University Hospital Zurich: Helena Aebersold, Fabienne Foster, Matthias Schwenkglenks.

Medical Image Analysis Center AG Basel: Marco Düring, Tim Sinnecker, Anna Altermatt, Michael Amann, Petra Huber, Manuel Hürbin, Esther Ruberte, Alain Thöni, Jens Würfel, Vanessa Zuber.

Department Clinical research, University of Basel: Michael Coslovsky (Head), Pia Neuschwander, Patrick Simon, Olivia Wunderlin.

Schiller AG Baar: Ramun Schmid, Christian Baumann.

# Supplementary Tables

| **Table S1. Blood pressure classification (20)** | | | |
| --- | --- | --- | --- |
| **Category** | **Systolic (mmHg)** |  | **Diastolic (mmHg)** |
|  |  |  |  |
| **Optimal** | <120 | and | <80 |
| **Normal** | 120–129 | and/or | 80–84 |
| **High normal** | 130–139 | and/or | 85–89 |
| **Hypertension grade 1** | 140–159 | and/or | 90–99 |
| **Hypertension grade 2 and 3** | ≥ 160 | and/or | ≥100 |
|  | | | |
| BP indicates blood pressure. BP categorized according to current guidelines of the European Society of Hypertension (20). The highest BP level (systolic or diastolic) defines the BP category. | | | |

| Table S2. Comparison baseline characteristics of patients with and without MRI at baseline and after 2 years | | | | | | |
| --- | --- | --- | --- | --- | --- | --- |
|  | **Study Population** | | **Patients without MRI** |  |  | |
|  | n = 1213 | | n = 911 | P value | Missing | |
| Age, years | 71.4 (±8.4) | | 74.7 (±8.1) | <0.001 |  | |
| Sex, male, % | 897 (73.9) | | 653 (71.7) | 0.26 |  | |
| Body mass index, kg/m^2^ | 27.7 (±4.7) | | 27.7 (±4.8) | 0.90 |  | |
| Education, years | 13.3 (±3) | | 12.7 (±3) | <0.001 |  | |
| Geriatric depression scale, points | 1 [0, 2] | | 1 [0, 3] | <0.001 |  | |
| Active smoking, % | 84 (6.9) | | 71 (7.8) | 0.54 |  | |
| Regular physical active, % | 634 (52.3) | | 392 (43.1) | <0.001 |  | |
| Heart rate, bpm | 66 [57, 77] | | 65 [60, 74] | 0.98 |  | |
| Systolic blood pressure, mmHg | 135 (±18) | | 133 (±19) | 0.006 |  | |
| Diastolic blood pressure, mmHg | 79 (±12) | | 76 (±11) | <0.001 |  | |
| Blood pressure categories, % |  | |  | 0.036 |  | |
| Optimal | 211 (17.4) | | 208 (22.8) |  |  | |
| Normal | 249 (20.5) | | 181 (19.9) |  |  | |
| High normal | 265 (21.8) | | 186 (20.4) |  |  | |
| Hypertension grade 1 | 348 (28.7) | | 246 (27.0) |  |  | |
| Hypertension grade 2 and 3 | 140 (11.5) | | 90 (9.9) |  |  | |
| Atrial fibrillation type, % |  | |  | <0.001 |  | |
| Paroxysmal | 567 (46.7) | | 397 (43.6) |  |  | |
| Non-paroxysmal | 646 (53.3) | | 514 (56.4) |  |  | |
| Heart rhythm at the baseline visit, % |  | |  | <0.001 | 0.4 | |
| Sinus rhythm | 673 (55.7) | | 327 (36.1) |  |  | |
| Atrial Fibrillation | 408 (33.7) | | 378 (41.7) |  |  | |
| Atrial Flutter | 43 (3.6) | | 22 (2.4) |  |  | |
| Other | 85 (7.0) | | 179 (19.8) |  |  | |
| CHA_2_DS_2_-VASc Score, points | 3.1 (±1.7) | | 3.8 (±1.6) | <0.001 | 0.1 | |
| History of arterial hypertension, % | 821 (67.7) | | 638 (70.0) | 0.27 |  | |
| Antihypertensive treatment, % | 792 (65.3) * | | 620 (68.1) ^†^ | 0.20 |  | |
| Number of antihypertensive drugs, % |  | |  | <0.001 | 33.5 | |
| 1 | 142 (17.9) | | 86 (13.9) |  |  | |
| 2 | 277 (35.0) | | 168 (27.1) |  |  | |
| ≥3 | 373 (47.1) | | 366 (59.0) |  |  | |
| History of heart failure, % | 228 (18.8) | | 299 (32.9) | <0.001 |  | |
| History of diabetes mellitus, % | 173 (14.3) | | 167 (18.3) | 0.013 |  | |
| History of stroke or TIA, % | 232 (19.1) | | 189 (20.8) | 0.38 |  | |
| History of coronary heart disease, % | 312 (25.7) | | 331 (36.3) | <0.001 |  | |
| History of renal failure, % | 176 (14.5) | | 235 (25.9) | <0.001 | 0.1 | |
| History of systemic embolism, % | 52 (4.3) | | 58 (6.4) | 0.041 |  | |
| History of peripheral artery disease, % | 64 (5.3) | | 89 (9.8) | <0.001 |  | |
| History of OSAS, % | 157 (12.9) | | 150 (16.5) | 0.026 |  | |
| Hypercholesterolemia, % | 325 (28.4) | | 213 (25.2) | 0.13 | 6.4 | |
| Oral anticoagulation, % | 1086 (89.5) | | 830 (91.1) | 0.26 |  | |
| Antiplatelet therapy, % | 208 (17.2) | | 215 (23.6) | <0.001 | 0.1 | |
| **Cognitive testing** |  |  |  |  |  |  |
| MoCA score, points | 26 [24, 28] | | 26 [23, 27] | <0.001 |  | |
| Semantic Fluency Test, points | 20 [16, 23] | | 18 [15, 22] | <0.001 |  | |
| Digit Symbol Substitution Test, points | 47 [37, 56] | | 40 [33, 50] | <0.001 |  | |
|  |  |  |  |  |  | |
| Data are presented as n (%), mean (±standard deviation), or median [interquartile range]. BP = blood pressure; MoCA = Montreal Cognitive Assessment; OSAS = obstructive sleep apnea syndrome; and TIA = transient ischemic attack.  Optimal BP indicates systolic BP <120 and diastolic BP <80mmHg; Normal BP, systolic BP 120-129 mmHg and/or diastolic BP 80-84 mmHg; High normal BP, systolic BP 130-139 mmHg and/or diastolic BP 85-89 mmHg; Hypertension grade 1, systolic BP 140-159 mmHg and/or diastolic BP 90-99 mmHg; Hypertension grade 2 and 3, systolic BP ≥160 and/or diastolic BP ≥100 mmHg.  ^*^Of the 821 patients with hypertension, 792 (96.5%) were on antihypertensive treatment.  ^†^Of the 638 patients with hypertension, 620 (97.2%) were on antihypertensive treatment. | | | | | | |

| Table S3. Results of cognitive testing at baseline stratified by blood pressure categories | | | | | |
| --- | --- | --- | --- | --- | --- |
|  | **MoCA** | | **SFT** | | **DSST** |
|  | points | | points | | points |
| **Overall** | 26 [24, 28] | | 20 [16, 23] | | 47 [37, 56] |
|  |  |  | |  | |
| **Blood pressure categories** |
| Optimal BP | 26 [25, 28] | | 20 [16, 24] | | 47 [39, 55] |
| Normal BP | 26 [24, 28] | | 19 [16, 23] | | 47 [36, 57] |
| High normal BP | 26 [25, 28] | | 20 [16, 23] | | 47 [38, 56] |
| Hypertension grade 1 | 26 [24, 28] | | 20 [16, 23] | | 47 [38, 57] |
| Hypertension grade 2 and 3 | 26 [24, 28] | | 19 [16, 23] | | 44 [36, 55] |
| P value | 0.11 | | 0.29 | | 0.45 |
|  | | | | | |
| Data are median [interquartile range]. BP = blood pressure; DSST = Digit Symbol Substitution Test; MoCA = Montreal Cognitive Assessment; and SFT = Semantic Fluency Test. Groups were compared using the Kruskal-Wallis Tests.  Optimal BP indicates systolic BP <120 and diastolic BP <80 mmHg; Normal BP, systolic BP 120-129 mmHg and/or diastolic BP 80-84 mmHg; High normal BP, systolic BP 130-139 mmHg and/or diastolic BP 85-89 mmHg; Hypertension grade 1, systolic BP 140-159 mmHg and/or diastolic BP 90-99 mmHg; Hypertension grade 2 and 3, systolic BP ≥160 and/or diastolic BP ≥100 mmHg. n = 1213. | | | | | |

| Table S4. Association between blood pressure with new ischemic lesions and new white matter lesions | | | | |
| --- | --- | --- | --- | --- |
|  | **Incidence** | | **Volume** | |
|  | Model 1 | Model 2 | Model 1 | Model 2 |
|  | OR (95% CI) | OR (95% CI) | β-coefficient (95% CI) | β-coefficient (95% CI) |
| **Ischemic Lesions** | n= 1213 | | n= 66 | |
| **Systolic BP**, per 10 mmHg | 1.06 (0.92 to 1.22), *p*=0.42 | 1.10 (0.95 to 1.27), *p*=0.21 | -0.05 (-0.26 to 0.16), *p*=0.62 | 0.11 (-0.13 to 0.35), *p*=0.37 |
|  | | | | |
| **Diastolic BP**, **per** 10 mmHg | 1.15 (0.94 to 1.41), *p*=0.17 | 1.23 (0.99 to 1.52), *p*=0.06 | -0.22 (-0.52 to 0.08), *p*=0.15 | -0.01 (-0.34 to 0.32), *p*=0.94 |
|  | | | | |
| **Blood pressure categories** | | | | |
| Optimal | *reference* | *reference* | *reference* | *reference* |
| Normal | 1.10 (0.48 to 2.62) | 1.09 (0.47 to 2.65) | -0.15 (-1.51 to 1.21) | 0.37(-1.15 to 1.90) |
| High normal | 1.08 (0.47 to 2.54) | 1.12 (0.49 to 2.69) | -0.59 (-1.94 to 0.77) | 0.37 (-1.15 to 1.89) |
| Hypertension grade 1 | 0.82 (0.36 to 1.92) | 0.85 (0.37 to 2.06) | -1.32 (-2.66 to 0.01) | -0.50 (-2.01 to 1.02) |
| Hypertension grade 2 and 3 | 1.71 (0.71 to 4.19) | 2.15 (0.86 to 5.48) | -0.44 (-1.86 to 0.99) | 0.84 (-0.88 to 2.56) |
| P for linear trend | 0.43 | 0.21 | 0.19 | 0.67 |
| P for quadratic trend | 0.35 | 0.23 | 0.31 | 0.57 |
|  | | | | |
| **White Matter Lesions** | n= 1213 | | n= 223 | |
| **Systolic BP**, per 10 mmHg | 0.96 (0.88 to 1.04), *p*=0.35 | 0.96 (0.88 to 1.04), *p*=0.32 | -0.02 (-0.12 to 0.08), *p*=0.64 | -0.02 (-0.12 to 0.09), *p*=0.78 |
|  | | | | |
| **Diastolic BP**, **per** 10 mmHg | 1.06 (0.93 to 1.19), *p*=0.39 | 1.06 (0.93 to 1.20), *p*=0.40 | 0.06 (-0.09 to 0.21), *p*=0.41 | 0.07 (-0.08 to 0.23), *p*=0.37 |
|  | | | | |
| **Blood pressure categories** | | | | |
| Optimal | *reference* | *reference* | *reference* | *reference* |
| Normal | 1.54 (0.95 to 2.51) | 1.47 (0.90 to 2.42) | 0.07 (-0.50 to 0.65) | 0.03 (-0.56 to 0.62) |
| High normal | 1.15 (0.71 to 1.90) | 1.15 (0.70 to 1.90) | -0.43 (-1.02 to 0.16) | -0.38 (-0.99 to 0.22) |
| Hypertension grade 1 | 1.19 (0.75 to 1.92) | 1.15 (0.72 to 1.88) | -0.22 (-0.78 to 0.34) | -0.21 (-0.79 to 0.37) |
| Hypertension grade 2 and 3 | 1.13 (0.63 to 2.01) | 1.12 (0.62 to 2.02) | 0.07 (-0.63 to 0.76) | 0.09 (- 0.62 to 0.81) |
| P for linear trend | 0.99 | 0.99 | 0.83 | 0.95 |
| P for quadratic trend | 0.36 | 0.43 | 0.17 | 0.18 |
|  | | | | |
| Model 1 was adjusted for age and sex. Model 2 was additionally adjusted for education, body mass index, smoking status, previous stroke or transient ischemic attack, history of diabetes, history of heart failure, history of coronary heart disease, atrial fibrillation type, oral anticoagulation, antithrombotic treatment, and antihypertensive treatment.  BP = blood pressure; CI = confidence interval; and OR = odds ratio.  Optimal BP indicates systolic BP <120 and diastolic BP <80 mmHg; Normal BP, systolic BP 120-129 mmHg and/or diastolic BP 80-84 mmHg; High normal BP, systolic BP 130-139 mmHg and/or diastolic BP 85-89 mmHg; Hypertension grade 1, systolic BP 140-159 mmHg and/or diastolic BP 90-99 mmHg; Hypertension grade 2 and 3, systolic BP ≥160 and/or diastolic BP ≥100 mmHg. | | | | |

| Table S5. Association between blood pressure with new microbleeds and composite endpoint | | | | | |
| --- | --- | --- | --- | --- | --- |
|  | **Microbleeds** | | **Composite Endpoint*** | | |
|  | Incidence | | Incidence | | |
|  | Model 1 | Model 2 | Model 1 | Model 2 | |
|  | OR (95% CI) | OR (95% CI) | OR (95% CI) | OR (95% CI) | |
|  | n= 1174 | | n= 1185 | | |
| **Systolic BP**, per 10 mmHg | 1.01 (0.91 to 1.11), *p*=0.88 | 1.03 (0.93 to 1.14), *p*=0.54 | 0.99 (0.92 to 1.06), *p*=0.69 | 0.99 (0.92 to 1.07), *p*=0.88 | |
| **Diastolic BP**, per 10 mmHg | 1.11 (0.95 to 1.29), *p*=0.18 | 1.13 (0.97 to 1.32), *p*=0.12 | 1.11 (1.00 to 1.24), *p*=0.05 | 1.12 (1.00 to 1.26), *p*=0.04 | |
|  |  |  |  | |  |
| **Blood pressure categories** | | | | | |
| Optimal | *reference* | *reference* | *reference* | *reference* | |
| Normal | 1.26 (0.70 to 2.29) | 1.37 (0.75 to 2.53) | 1.49 (0.98 to 2.29) | 1.48 (0.96 to 2.29) | |
| High normal | 0.84 (0.45 to 1.57) | 0.90 (0.48 to 1.71) | 1.08 (0.70 to 1.67) | 1.09 (0.71 to 1.71) | |
| Hypertension grade 1 | 1.10 (0.63 to 1.96) | 1.21 (0.68 to 2.18) | 1.25 (0.84 to 1.89) | 1.26 (0.84 to 1.92) | |
| Hypertension grade 2 and 3 | 1.11 (0.55 to 2.20) | 1.36 (0.66 to 2.73) | 1.18 (0.72 to 1.95) | 1.27 (0.76 to 2.12) | |
| P for linear trend | 0.91 | 0.53 | 0.76 | 0.55 | |
| P for quadratic trend | 0.78 | 0.72 | 0.47 | 0.61 | |
|  | | | | | |
| Model 1 was adjusted for age and sex. Model 2 was additionally adjusted for education, body mass index, smoking status, previous stroke or transient ischemic attack, history of diabetes, history of heart failure, history of coronary heart disease, atrial fibrillation type, oral anticoagulation, antithrombotic treatment, and antihypertensive treatment. BP = blood pressure; CI = confidence interval; and OR = odds ratio.  *Composite endpoint for microvascular damage of white matter lesions, microbleeds and small non-cortical infarcts.  Optimal BP indicates systolic BP <120 and diastolic BP <80 mmHg; Normal BP, systolic BP 120-129 mmHg and/or diastolic BP 80-84 mmHg; High normal BP, systolic BP 130-139 mmHg and/or diastolic BP 85-89 mmHg; Hypertension grade 1, systolic BP 140-159 mmHg and/or diastolic BP 90-99 mmHg; Hypertension grade 2 and 3, systolic BP ≥160 and/or diastolic BP ≥100 mmHg. | | | | | |

| Table S6. Interaction analysis for the association of blood pressure with new ischemic lesions and new white matter lesions | |
| --- | --- |
|  | **Incidence** |
| **New Ischemic Lesions** |  |
| **Atrial fibrillation type** | ***p* for interaction = 0.05** |
| Paroxysmal | 0.92 (0.72 to 1.17), *p*=0.52 |
| Non-Paroxysmal | 1.24 (1.02 to 1.51), *p*=0.03 |
|  | |
| **Heart Rhythm** | ***p* for interaction = 0.62** |
| AF and Atrial Flutter | 1.17 (0.95 to 1.43), *p*=0.13 |
| Sinus Rhythm | 1.06 (0.83 to 1.35), *p*=0.61 |
|  |  |
| **New White Matter Lesions** |  |
| **Atrial fibrillation type** | ***p* for interaction = 0.26** |
| Paroxysmal | 0.91 (0.80 to 1.04), *p*=0.17 |
| Non-Paroxysmal | 0.99 (0.89 to 1.11), *p*=0.92 |
|  | |
| **Heart Rhythm** | ***p* for interaction = 0.65** |
| AF and Atrial Flutter | 0.98 (0.86 to 1.12), *p*=0.82 |
| Sinus Rhythm | 0.93 (0.82 to 1.06), *p*=0.28 |
|  |  |
| Data are p values for interaction and odds ratio (95% confidence interval). AF = atrial fibrillation. Models are adjusted for age, sex, education, body mass index, smoking status, previous stroke or transient ischemic attack, history of diabetes, history of heart failure, history of coronary heart disease, atrial fibrillation type, oral anticoagulation, antithrombotic treatment, and antihypertensive treatment. Predictor variable: systolic blood pressure (per 10 mmHg). AF type (paroxysmal vs non paroxysmal) and heart rhythm (AF and atrial flutter vs sinus rhythm) were compared using Analysis of Variance (ANOVA).  Valid n for paroxysmal = 567. Valid n for non-paroxysmal = 646.  Valid n for AF and atrial flutter = 451. Valid n for sinus rhythm = 673. | |

| Table S7. Sensitivity analysis: Association between blood pressure and cognitive decline in patients without study termination | | | | | | |
| --- | --- | --- | --- | --- | --- | --- |
|  | **Number of events** | **Patient-years** | | **Incidence rate per 100 patient-years** | **Cox Regression Model 1**  HR (95% CI), *p* value | **Cox Regression Model 2** HR (95% CI), *p* value |
| **MoCA** | 95 | 4477 | | 2.12 |  | |
| **Systolic BP**, per 10 mmHg | | | | | 0.97 (0.86 to 1.08), *p*=0.55 | 0.96 (0.86 to 1.08), *p*=0.54 |
| **Diastolic BP**, per 10 mmHg | | | | | 0.93 (0.78 to 1.11), *p*=0.42 | 0.92 (0.77 to 1.11), *p*=0.39 |
|  | | | | |  |  |
| **Blood pressure categories** | | | | | | |
| Optimal | 15 | 764 | | 1.96 | *reference* | *reference* |
| Normal | 15 | 921 | | 1.63 | 0.76 (0.37 to 1.55), *p*=0.45 | 0.74 (0.36 to 1.52), *p*=0.41 |
| High normal | 30 | 970 | | 3.09 | 1.42 (0.76 to 2.64), *p*=0.27 | 1.36 (0.72 to 2.57), *p*=0.34 |
| Hypertension grade 1 | 25 | 1315 | | 1.14 | 0.85 (0.45 to 1.62), *p*=0.63 | 0.84 (0.44 to 1.62), *p*=0.61 |
| Hypertension grade 2 and 3 | 10 | 507 | | 1.97 | 0.86 (0.39 to 1.93), *p*=0.72 | 0.82 (0.36 to 1.85), *p*=0.63 |
| p for linear trend |  |  | |  | 0.85 | 0.77 |
| p for quadratic trend |  |  | |  | 0.56 | 0.58 |
|  | | | | | | |
| **SFT** | 315 | | 5357 | 5.88 |  | |
| **Systolic BP**, per 10 mmHg | | | | | 0.95 (0.89 to 1.01), *p*=0.11 | 0.95 (0.89 to 1.01), *p*=0.11 |
| **Diastolic BP**, per 10 mmHg | | | | | 0.95 (0.86 to 1.04), *p*=0.27 | 0.95 (0.86 to 1.05), *p*=0.28 |
|  | | | | |  |  |
| **Blood pressure categories** | | | | | | |
| Optimal | 70 | | 906 | 7.73 | *reference* | *reference* |
| Normal | 70 | | 1074 | 6.52 | 0.83 (0.60 to 1.16), *p*=0.28 | 0.81 (0.58 to 1.14), *p*=0.23 |
| High normal | 61 | | 1178 | 5.18 | 0.67 (0.47 to 0.94), *p*=0.021 | 0.66 (0.46 to 0.93), *p*=0.017 |
| Hypertension grade 1 | 77 | | 1622 | 4.75 | 0.60 (0.44 to 0.84), *p*=0.002 | 0.60 (0.43 to 0.84), *p*=0.003 |
| Hypertension grade 2 and 3 | 37 | | 577 | 6.41 | 0.82 (0.55 to 1.22), *p*=0.33 | 0.79 (0.52 to 1.19), *p*=0.26 |
| P for linear trend |  | |  |  | 0.10 | 0.09 |
| P for quadratic trend |  | |  |  | 0.031 | 0.035 |
|  | | | | | | |
| **DSST** | 84 | | 4471 | 1.88 |  | |
| **Systolic BP**, per 10 mmHg | | | | | 0.93 (0.82 to 1.06), *p*=0.27 | 0.92 (0.81 to 1.05), *p*=0.21 |
| **Diastolic BP**, per 10 mmHg | | | | | 0.96 (0.80 to 1.16), *p*=0.67 | 0.95 (0.79 to 1.15), *p*=0.62 |
|  | | | | |  |  |
| **Blood pressure categories** | | | | | | |
| Optimal | 15 | | 758 | 1.98 | *reference* | *reference* |
| Normal | 21 | | 902 | 2.33 | 1.19 (0.61 to 2.32), *p*=0.60 | 1.18 (0.60 to 2.30), *p*=0.64 |
| High normal | 21 | | 995 | 2.11 | 1.06 (0.55 to 2.07), *p*=0.86 | 1.00 (0.51 to 1.96), *p*=1.00 |
| Hypertension grade 1 | 16 | | 1316 | 1.22 | 0.61 (0.30 to 1.23), *p*=0.16 | 0.57 (0.28 to 1.16), *p*=0.12 |
| Hypertension grade 2 and 3 | 11 | | 500 | 2.20 | 1.15 (0.53 to 2.52), *p*=0.72 | 1.08 (0.49 to 2.40), *p*=0.85 |
| P for linear trend |  | |  |  | 0.65 | 0.52 |
| P for quadratic trend |  | |  |  | 0.61 | 0.56 |
|  | | | | | | |

Decline of cognitive function was defined as a decrease of one age-education normalized standard deviation from each patient’s test baseline value. Model 1 was adjusted for age, sex, and education. n = 1026.

Model 2 was additionally adjusted for geriatric depression score, body mass index, smoking status, previous stroke or transient ischemic attack, history of diabetes, history of heart failure, history of coronary heart disease, atrial fibrillation type, oral anticoagulation, antithrombotic treatment, and antihypertensive treatment. n = 1025.

BP = blood pressure; CI = confidence interval; DSST = digit symbol substitution test; HR = hazard ratio; MoCA = Montreal Cognitive Assessment; and SFT = semantic fluency test.

Optimal BP indicates systolic BP <120 and diastolic BP <80 mmHg; Normal BP, systolic BP 120-129 mmHg and/or diastolic BP 80-84 mmHg; High normal BP, systolic BP 130-139 mmHg and/or diastolic BP 85-89 mmHg; Hypertension grade 1, systolic BP 140-159 mmHg and/or diastolic BP 90-99 mmHg; Hypertension grade 2 and 3, systolic BP ≥160 and/or diastolic BP ≥100 mmHg.

| Table S8. Sensitivity analysis: Association between blood pressure and cognitive decline including patients without MRI data | | | | | |
| --- | --- | --- | --- | --- | --- |
|  | **Number of events** | **Patient-years** | **Incidence rate per 100 patient-years** | **Cox Regression Model 1**  HR (95% CI), *p* value | **Cox Regression Model 2**  HR (95% CI), *p* value |
| **MoCA** | 240 | 7766 | 3.09 |  | |
| **Systolic BP**, per 10 mmHg | | | | 0.96 (0.89 to 1.03), *p*=0.25 | 0.96 (0.90 to 1.03), *p*=0.29 |
| **Diastolic BP**, per 10 mmHg | | | | 0.89 (0.80 to 1.00), *p*=0.043 | 0.89 (0.79 to 1.00), *p*=0.046 |
|  | | | | | |
| **Blood pressure categories** | | | | | |
| Optimal | 47 | 1475 | 3.19 | *reference* | *reference* |
| Normal | 48 | 1608 | 2.99 | 0.87 (0.58 to 1.30), *p*=0.49 | 0.88 (0.58 to 1.32), *p*=0.53 |
| High normal | 55 | 1653 | 3.33 | 0.95 (0.64 to 1.40), *p*=0.78 | 0.95 (0.64 to 1.42), *p*=0.81 |
| Hypertension grade 1 | 66 | 2185 | 3.02 | 0.83 (0.57 to 1.20), *p*=0.32 | 0.83 (0.57 to 1.22), *p*=0.34 |
| Hypertension grade 2 and 3 | 24 | 845 | 2.84 | 0.79 (0.48 to 1.29), *p*=0.34 | 0.79 (0.48 to 1.31), *p*=0.37 |
| P for linear trend |  |  |  | 0.33 | 0.35 |
| P for quadratic trend |  |  |  | 0.95 | 0.93 |
|  | | | | | |
| **SFT** | 602 | 9558 | 6.30 |  | |
| **Systolic BP**, per 10 mmHg | | | | 0.94 (0.90 to 0.99), *p*=0.009 | 0.95 (0.90 to 0.99), *p*=0.016 |
| **Diastolic BP**, per 10 mmHg | | | | 0.91 (0.85 to 0.98), *p*=0.011 | 0.91 (0.85 to 0.98), *p*=0.014 |
|  | | | | | |
| **Blood pressure categories** | | | | | |
| Optimal | 140 | 1793 | 7.81 | *reference* | *reference* |
| Normal | 129 | 1889 | 6.83 | 0.87 (0.68 to 1.10), *p*=0.25 | 0.87 (0.68 to 1.11), *p*=0.25 |
| High normal | 123 | 2075 | 5.93 | 0.74 (0.58 to 0.95), *p*=0.017 | 0.74 (0.58 to 0.95), *p*=0.017 |
| Hypertension grade 1 | 152 | 2776 | 5.48 | 0.68 (0.54 to 0.85), *p* <0.001 | 0.68 (0.54 to 0.86), *p*=0.001 |
| Hypertension grade 2 and 3 | 58 | 1024 | 5.66 | 0.70 (0.52 to 0.95), *p*=0.024 | 0.70 (0.52 to 0.96), *p*=0.028 |
| P for linear trend |  |  |  | 0.005 | 0.006 |
| P for quadratic trend |  |  |  | 0.28 | 0.27 |
|  | | | | | |
| **DSST** | 205 | 7709 | 2.66 |  | |
| **Systolic BP**, per 10 mmHg | | | | 0.96 (0.89 to 1.04), *p*=0.31 | 0.96 (0.89 to 1.04), *p*=0.28 |
| **Diastolic BP**, per 10 mmHg | | | | 0.98 (0.87 to 1.10), *p*=0.74 | 0.99 (0.88 to 1.12), *p*=0.91 |
|  | | | | | |
| **Blood pressure categories** | | | | | |
| Optimal | 38 | 1473 | 2.58 | *reference* | *reference* |
| Normal | 50 | 1565 | 3.19 | 1.21 (0.79 to 1.84), *p*=0.38 | 1.16 (0.76 to 1.78), *p*=0.49 |
| High normal | 47 | 1650 | 2.85 | 1.07 (0.70 to 1.64), *p*=0.76 | 1.07 (0.70 to 1.65), *p*=0.75 |
| Hypertension grade 1 | 48 | 2196 | 2.19 | 0.80 (0.52 to 1.23), *p*=0.32 | 0.80 (0.52 to 1.23), *p*=0.30 |
| Hypertension grade 2 and 3 | 22 | 825 | 2.67 | 0.99 (0.58 to 1.68), *p*=0.97 | 0.96 (0.56 to 1.63), *p*=0.87 |
| P for linear trend |  |  |  | 0.46 | 0.43 |
| P for quadratic trend |  |  |  | 0.84 | 0.81 |
|  | | | | | |
| Decline of cognitive function was defined as a decrease of one age-education normalized standard deviation from each patient’s test baseline value. Model 1 was adjusted for age, sex, and education; n = 2124. Model 2 was additionally adjusted for geriatric depression score, body mass index, smoking status, previous stroke or transient ischemic attack, history of diabetes, history of heart failure, history of coronary heart disease, atrial fibrillation type, oral anticoagulation, antithrombotic treatment, and antihypertensive treatment; n = 2119. BP = blood pressure; CI = confidence interval; DSST = digit symbol substitution test; HR= hazard ratio; MoCA = Montreal Cognitive Assessment; and SFT = semantic fluency test. Optimal BP indicates systolic BP <120 and diastolic BP <80 mmHg; Normal BP, systolic BP 120-129 mmHg and/or diastolic BP 80-84 mmHg; High normal BP, systolic BP 130-139 mmHg and/or diastolic BP 85-89 mmHg; Hypertension grade 1, systolic BP 140-159 mmHg and/or diastolic BP 90-99 mmHg; Hypertension grade 2 and 3, systolic BP ≥160 and/or diastolic BP ≥100 mmHg. | | | | | |

| Table S9. Sensitivity analysis: Association between blood pressure and cognitive decline additionally adjusted for regular physical activity and hypercholesterolemia | | | |
| --- | --- | --- | --- |
|  | **Cox Regression Model 1**  HR (95% CI), *p* value | **Cox Regression Model 2**  HR (95% CI), *p* value | **Cox Regression Model 3**  HR (95% CI), *p* value |
| **MoCA** |  |  |  |
| **Systolic BP**, per 10 mmHg | 0.94 (0.85 to 1.03), *p*=0.19 | 0.94 (0.85 to 1.04), *p*=0.23 | 0.95 (0.86 to 1.05), *p*=0.34 |
| **Diastolic BP**, per 10 mmHg | 0.87 (0.75 to 1.02), *p*=0.08 | 0.87 (0.74 to 1.02), *p*=0.08 | 0.88 (0.75 to 1.03), *p*=0.11 |
|  |  |  |  |
| **Blood pressure categories** |  |  |  |
| Optimal | *reference* | *reference* | *reference* |
| Normal | 0.86 (0.48 to 1.55), *p*=0.62 | 0.85 (0.47 to 1.53), *p*=0.59 | 0.86 (0.46 to 1.60), *p*=0.64 |
| High normal | 1.19 (0.69 to 2.04), *p*=0.53 | 1.19 (0.69 to 2.07), *p*=0.53 | 1.29 (0.73 to 2.29), *p*=0.38 |
| Hypertension grade 1 | 0.74 (0.43 to 1.29), *p*=0.29 | 0.76 (0.43 to 1.34), *p*=0.34 | 0.82 (0.46 to 1.48), *p*=0.51 |
| Hypertension grade 2 and 3 | 0.77 (0.39 to 1.54), *p*=0.46 | 0.75 (0.37 to 1.53), *p*=0.43 | 0.85 (0.41 to 1.75), *p*=0.65 |
| P for linear trend | 0.37 | 0.38 | 0.63 |
| P for quadratic trend | 0.61 | 0.57 | 0.56 |
|  |  |  |  |
| **SFT** |  |  |  |
| **Systolic BP**, per 10 mmHg | 0.95 (0.89 to 1.00), *p*=0.07 | 0.94 (0.89 to 1.00), *p*=0.06 | 0.95 (0.89 to 1.01), *p*=0.07 |
| **Diastolic BP**, per 10 mmHg | 0.95 (0.87 to 1.04), *p*=0.27 | 0.95 (0.87 to 1.04), *p*=0.30 | 0.97 (0.88 to 1.06), *p*=0.52 |
|  |  |  |  |
| **Blood pressure categories** |  |  |  |
| Optimal | *reference* | *reference* | *reference* |
| Normal | 0.78 (0.57 to 1.06), *p*=0.11 | 0.75 (0.55 to 1.03), *p*=0.07 | 0.81 (0.59 to 1.12), *p*=0.21 |
| High normal | 0.64 (0.47 to 0.88), *p*=0.005 | 0.63 (0.46 to 0.87), *p*=0.005 | 0.66 (0.48 to 0.92), *p*=0.02 |
| Hypertension grade 1 | 0.58 (0.43 to 0.78), *p* <0.001 | 0.57 (0.42 to 0.77), *p* <0.001 | 0.59 (0.43 to 0.82), *p*=0.001 |
| Hypertension grade 2 and 3 | 0.76 (0.52 to 1.10), *p*=0.15 | 0.72 (0.49 to 1.05), *p*=0.09 | 0.78 (0.53 to 1.14), *p*=0.20 |
| P for linear trend | 0.039 | 0.025 | 0.055 |
| P for quadratic trend | 0.015 | 0.020 | 0.033 |
|  |  |  |  |
| **DSST** |  |  |  |
| **Systolic BP**, per 10 mmHg | 0.95 (0.85 to 1.05), *p*=0.30 | 0.94 (0.84 to 1.05), *p*=0.27 | 0.94 (0.84 to 1.05), *p*=0.26 |
| **Diastolic BP**, per 10 mmHg | 0.95 (0.81 to 1.12), *p*=0.56 | 0.97 (0.83 to 1.14), *p*=0.73 | 0.97 (0.82 to 1.14), *p*=0.68 |
|  |  |  |  |
| **Blood pressure categories** |  |  |  |
| Optimal | *reference* | *reference* | *reference* |
| Normal | 1.16 (0.66 to 2.07), *p*=0.60 | 1.16 (0.65 to 2.06), *p*=0.62 | 1.14 (0.63 to 2.06), *p*=0.67 |
| High normal | 1.00 (0.56 to 1.79), *p*=0.998 | 1.00 (0.56 to 1.79), *p*=0.999 | 0.98 (0.54 to 1.79), *p*=0.95 |
| Hypertension grade 1 | 0.79 (0.44 to 1.40), *p*=0.42 | 0.78 (0.43 to 1.40), *p*=0.40 | 0.76 (0.42 to 1.39), *p*=0.37 |
| Hypertension grade 2 and 3 | 0.96 (0.48 to 1.94), *p*=0.92 | 0.95 (0.47 to 1.94), *p*=0.89 | 0.92 (0.45 to 1.90), *p*=0.83 |
| P for linear trend | 0.54 | 0.52 | 0.47 |
| P for quadratic trend | 0.99 | 0.99 | 0.98 |
|  |  |  |  |
| Decline of cognitive function was defined as a decrease of one age-education normalized standard deviation from each patient’s test baseline value. Model 1 was adjusted for age, sex, and education; n = 1213.  Model 2 was additionally adjusted for geriatric depression score, body mass index, smoking status, previous stroke or transient ischemic attack, history of diabetes, history of heart failure, history of coronary heart disease, atrial fibrillation type, oral anticoagulation, antithrombotic treatment, and antihypertensive treatment; n=1212  Model 3 was additionally adjusted for regular physical activity, and hypercholesterolemia; n = 1144.  BP = blood pressure; CI = confidence interval; DSST = digit symbol substitution test; HR= hazard ratio; MoCA = Montreal Cognitive Assessment; and SFT = semantic fluency test.  Optimal BP indicates systolic BP <120 and diastolic BP <80 mmHg; Normal BP, systolic BP 120-129 mmHg and/or diastolic BP 80-84 mmHg; High normal BP, systolic BP 130-139 mmHg and/or diastolic BP 85-89 mmHg; Hypertension grade 1, systolic BP 140-159 mmHg and/or diastolic BP 90-99 mmHg; Hypertension grade 2 and 3, systolic BP ≥160 and/or diastolic BP ≥100 mmHg. | | | |

| Table S10. Sensitivity Analysis: Mixed effects linear models for baseline blood pressure and cognitive function | | | | | |
| --- | --- | --- | --- | --- | --- |
|  |  | **Model 1** |  | **Model 2** |  |
|  |  | β-coefficient (95% CI) | *p*-value | β-coefficient (95% CI) | *p*-value |
| **MoCA** |  |  |  |  |  |
| **Continuous systolic BP** |  |  | Wald-type |  | Wald-type |
|  | Continuous SBP per 10 mmHg | 0.01 (-0.02 to 0.04) | *p*=0.42 | 0.01 (-0.02 to 0.04) | *p*=0.48 |
|  | Time | 0.03 (-0.09 to 0.14) | *p*=0.64 | 0.03 (-0.09 to 0.14) | *p*=0.66 |
|  | Interaction Continuous SBP : Time | 0.003 (-0.01 to 0.01) | *p*=0.45 | 0.003 (-0.005 to 0.01) | *p*=0.42 |
|  |  |  |  |  |  |
| **Continuous diastolic BP** |  |  | Wald-type |  | Wald-type |
|  | Continuous DBP, per 10 mmHg | 0.03 (-0.01 to 0.07) | *p*=0.20 | 0.02 (-0.02 to 0.06) | *p*=0.35 |
|  | Time | -0.01 (-0.11 to 0.09) | *p*=0.84 | -0.02 (-0.12 to 0.09) | *p*=0.77 |
|  | Interaction Continuous DBP : Time | 0.01 (-0.002 to 0.02) | *p*=0.11 | 0.01( -0.001 to 0.02) | *p*=0.08 |
|  |  |  |  |  |  |
| **Blood pressure categories** |  |  |  |  |  |
|  | LR test time x BP category |  | *p*=0.88 |  | *p*=0.88 |
|  |  |  |  |  |  |
|  | Time | 0.05 (0.01 to 0.10) |  | 0.06 (0.01 to 0.10) |  |
|  |  |  |  |  |  |
|  | Optimal | *reference* |  | *reference* |  |
|  |  |  |  |  |  |
|  | Normal | 0.02 (-0.13 to 0.17) |  | 0.04 ( -0.11 to 0.19) |  |
|  | Interaction Normal BP : Time | 0.01 (- 0.03 to 0.06) |  | 0.01 (-0.04 to 0.06) |  |
|  |  |  |  |  |  |
|  | High normal | 0.01 (-0.14 to 0.16) |  | 0.005 (-0.14 to 0.15) |  |
|  | Interaction High normal BP : Time | 0.02 (-0.03 to 0.06) |  | 0.02 (-0.03 to 0.06) |  |
|  |  |  |  |  |  |
|  | HTN 1° | 0.04 (-0.10 to 0.18) |  | 0.04 (-0.10 to 0.18) |  |
|  | Interaction HTN 1° : Time | 0.02 (-0.02 to 0.06) |  | 0.02 (-0.02 to 0.06) |  |
|  |  |  |  |  |  |
|  | HTN 2° and 3° | -0.006 (-0.18 to 0.17) |  | -0.002 (-0.18 to 0.18) |  |
|  | Interaction HTN 2° and 3° : Time | 0.03 (-0.03 to 0.08) |  | 0.03 (-0.03 to 0.08) |  |
|  |  |  |  |  |  |
| **SFT** |  |  |  |  |  |
| **Continuous systolic BP** |  |  | Wald-type |  | Wald-type |
|  | Continuous SBP per 10 mmHg | 0.02 (-0.01 to 0.05) | *p=*0.22 | 0.02 (-0.01 to 0.05) | *p=*0.26 |
|  | Time | 0.11 (-0.04 to 0.25) | *p*=0.15 | 0.11 (-0.04 to 0.25) | *p*=0.16 |
|  | Interaction Continuous SBP : Time | -0.004 (-0.01 to 0.01) | *p*=0.41 | -0.004 (-0.01 to 0.01) | *p=*0.45 |
|  |  |  |  |  |  |
| **Continuous diastolic BP** |  |  | Wald-type |  | Wald-type |
|  | Continuous DBP, per 10 mmHg | 0.03 (-0.01 to 0.08) | *p*=0.15 | 0.03 ( -0.02 to 0.08) | *p*=0.19 |
|  | Time | 0.07 (-0.06 to 0.20) | *p*=0.30 | 0.07 (-0.06 to 0.20) | *p*=0.30 |
|  | Interaction Continuous DBP : Time | -0.003 (-0.02 to 0.01) | *p*=0.73 | -0.002 (-0.02 to 0.01) | *p*=0.78 |
|  |  |  |  |  |  |
| **Blood pressure categories** |  |  |  |  |  |
|  | LR test time x BP category |  | *p*=0.44 |  | *p*=0.43 |
|  |  |  |  |  |  |
|  | Time | 0.07 (0.01 to 0.13) |  | 0.07 (0.02 to 0.13) |  |
|  |  |  |  |  |  |
|  | Optimal | *reference* |  | *reference* |  |
|  |  |  |  |  |  |
|  | Normal | 0.16 (-0.02 to 0.33) |  | 0.17 (-0.01 to 0.34) |  |
|  | Interaction Normal BP : Time | -0.03 (-0.09 to 0.03) |  | -0.03 (-0.09 to 0.03) |  |
|  |  |  |  |  |  |
|  | High normal | 0.11 (-0.06 to 0.29) |  | 0.10 (-0.07 to 0.28) |  |
|  | Interaction High Normal BP : Time | -0.01 (-0.07 to 0.05) |  | -0.01(-0.07 to 0.05) |  |
|  |  |  |  |  |  |
|  | HTN 1° | 0.19 (0.03 to 0.36) |  | 0.19 (0.02 to 0.36) |  |
|  | Interaction HTN1° : Time | -0.02 (-0.08 to 0.04) |  | -0.02 (-0.08 to 0.03) |  |
|  |  |  |  |  |  |
|  | HTN 2° and 3° | 0.20 ( -0.01 to 0.42) |  | 0.22 (0.005 to 0.43) |  |
|  | Interaction HTN 2° and 3° : Time | -0.07 (-0.14 to 0.01) |  | -0.06 (-0.13 to 0.01) |  |
|  |  |  |  |  |  |
| **DSST** |  |  |  |  |  |
| **Continuous systolic BP** |  |  | Wald-type |  | Wald-type |
|  | Continuous SBP per 10 mmHg | 0.01 (-0.01 to 0.03) | *p*=0.30 | 0.008 (-0.02 to 0.03) | *p*=0.51 |
|  | Time | 0.12 (0.02 to 0.21) | *p*=0.02 | 0.11 (0.02 to 0.21) | *p*=0.02 |
|  | Interaction Continuous SBP : Time | -0.004 (-0.01 to 0.003) | *p*=0.23 | -0.004 (-0.01 to 0.003) | *p*=0.26 |
|  |  |  |  |  |  |
| **Continuous diastolic BP** |  |  | Wald-type |  | Wald-type |
|  | Continuous DBP, per 10 mmHg | -0.03 ( -0.06 to 0.01) | *p*=0.10 | -0.03 ( -0.07 to 0.0003) | *p*=0.05 |
|  | Time | -0.02 (-0.11 to 0.06) | *p*=0.63 | -0.02 (-0.11 to 0.07) | *p*=0.64 |
|  | Interaction Continuous DBP : Time | 0.01 (-0.0003 to 0.02) | *p*=0.06 | 0.01 (-0.0002 to 0.02) | *p*=0.05 |
|  |  |  |  |  |  |
| **Blood pressure categories** |  |  |  |  |  |
|  | LR test time x BP category |  | *p*=0.68 |  | *p*=0.65 |
|  |  |  |  |  |  |
|  | Time | 0.08 (0.04 to 0.11) |  | 0.08 (0.04 to 0.11) |  |
|  |  |  |  |  |  |
|  | Optimal | *reference* |  | *reference* |  |
|  |  |  |  |  |  |
|  | Normal | 0.03 (-0.10 to 0.16) |  | 0.03 (-0.10 to 0.16) |  |
|  | Interaction Normal BP : Time | - 0.01 (-0.05 to 0.03) |  | -0.02 (-0.06 to 0.02) |  |
|  |  |  |  |  |  |
|  | High normal | 0.07 (-0.06 to 0.20) |  | 0.06 (-0.07 to 0.18) |  |
|  | Interaction High Normal BP : Time | -0.02 (-0.06 to 0.02) |  | -0.02 (-0.06 to 0.02) |  |
|  |  |  |  |  |  |
|  | HTN 1° | 0.13 (0.01 to 0.25) |  | 0.12 (-0.001 to 0.24) |  |
|  | Interaction HTN 1° : Time | -0.03 (-0.06 to 0.01) |  | -0.03 (-0.07 to 0.01) |  |
|  |  |  |  |  |  |
|  | HTN 2° and 3° | -0.02 (-0.17 to 0.13) |  | -0.04 (-0.19 to 0.12) |  |
|  | Interaction HTN 2° and 3° : Time | -0.01 (-0.06 to 0.04) |  | -0.01 (-0.06 to 0.04) |  |
|  |  |  |  |  |  |
| Model 1 was adjusted for age, sex, education, and baseline value. Model 2 was additionally adjusted for geriatric depression score, body mass index, smoking status, previous stroke or transient ischemic attack, diabetes, heart failure, coronary heart disease, atrial fibrillation type, oral anticoagulation, antithrombotic treatment, and antihypertensive treatment. BP = blood pressure; CI = confidence interval; DBP = diastolic blood pressure; HTN = hypertension; and SBP = systolic blood pressure. Time since the first measurement (random slope) and patient number nested within center (random intercept) were added to both models. Optimal BP indicates systolic BP <120 and diastolic BP <80mmHg; Normal BP, systolic BP ≥ 120 and <130mmHg or diastolic BP ≥ 80 and <85mmHg; High normal BP, systolic BP ≥ 130 and <140mmHg or diastolic BP ≥ 85 and <90mmHg; Hypertension grade 1, systolic BP ≥ 140 and <160mmHg or diastolic BP ≥ 90 and <100mmHg; Hypertension grade 2 and 3, systolic BP ≥ 160 or diastolic BP ≥ 100.  Number of observations n= 4275  P-values for models with continuous BP are from Wald-type tests for the hypotheses that the coefficients equal zero. For models with the interaction between BP-categories and time we provide a global likelihood ratio X2-test p-value for the interaction. | | | | | |

# Supplementary Figures

**Figure S1. Logistic regression models for incidence of new microbleeds and composite endpoint.**

Multivariable logistic and linear regression models adjusted for age, sex, education, body mass index, smoking status, previous stroke or transient ischemic attack, history of diabetes, history of heart failure, history of coronary heart disease, atrial fibrillation type, oral anticoagulation, antithrombotic treatment, and antihypertensive treatment. BP = blood pressure; CI = confidence interval; and OR = odds ratio.

*Composite endpoint of white matter lesions, microbleeds and small non cortical infarcts for microvascular damage.

Optimal BP indicates systolic BP <120 and diastolic BP <80 mmHg; Normal BP, systolic BP 120-129 mmHg and/or diastolic BP 80-84 mmHg; High normal BP, systolic BP 130-139 mmHg and/or diastolic BP 85-89 mmHg; Hypertension grade 1, systolic BP 140-159 mmHg and/or diastolic BP 90-99 mmHg; Hypertension grade 2 and 3, systolic BP ≥160 and/or diastolic BP ≥100 mmHg. Valid patients for microbleeds = 1174; Valid patients for composite endpoint = 1185.
